# Awakening immunity against cancer: a 2017 primer for clinicians

**DOI:** 10.1186/s40880-017-0233-4

**Published:** 2017-08-20

**Authors:** Amit Jain, Qing Zhang, Han-Chong Toh

**Affiliations:** 1 National Cancer Centre Singapore, 11 Hospital Drive, Singapore, 169610 Singapore; 2 GSR Ventures, Singapore, 048762 Singapore; 3 Tessa Therapeutics, Singapore, 038988 Singapore

**Keywords:** Immune checkpoint inhibitor, Chimeric antigen receptor T cells, Tumor microenvironment, Biomarker

## Abstract

Cancer immunotherapy has finally joined the pillars of cancer treatment—surgery, radiation, chemotherapy, hormonal therapy, and targeted therapy—in improving cancer patient lives. In the last 5 years, the development of immune checkpoint inhibitor and T cell therapy, particularly chimeric antigen receptor (CAR) T-cell therapy, has been remarkable for the speed, scale, and number of drug approvals. Still, these treatments may also bring unusual adverse effects and clinical outcomes including unprecedented long-term survival. Interrogating the tumor microenvironment and identifying better biomarkers hold the key to improving cancer immunotherapies. CAR T-cell therapy has dramatic effect on leukemias and lymphomas with significant cure rates, but has yet to show comparable effect on solid tumors. Cutting-edge technology will improve both processing and clinical effect of such therapies. Asia has the largest, most rapidly aging population and the largest number of cancer patients in the world. Research and development and clinical trial conduct of cancer immunotherapy in Asia remain nascent, but should be a crucial priority.

## A new horizon—learning from the giants

Cancer immunotherapy was announced as breakthrough of the year in 2013 on the premise and promise of immune checkpoint inhibitors, specifically T-cell-specific checkpoints, by the prestigious journal *Science* [1]. The last author of this editorial recalls his journey seeing the field grow through the eyes of giants, his mentors, Leslie Brent and Malcolm Brenner. Professor Leslie Brent had worked with his mentor Sir Peter Medawar to first describe acquired immune tolerance, for which Medawar won the Nobel Prize in Physiology or Medicine 1960. Professor Brent continued to push the frontiers of immune tolerance and coined the terms “adoptive cell transfer” and “graft versus host disease.” Malcolm Brenner, one of the true pioneers of cell and gene therapies and immunotherapy, introduced genetically modified T cells from bench to bedside to treat human disease for the first time [2]. These early visionaries epitomised a discipline for methodical, meticulous science in early days when these pioneers faced uphill battles with funding, sceptics, and frustrations.

James P. Allison is credited for introducing the concept of disinhibiting a T-cell checkpoint to facilitate clinically meaningful cancer responses [3]. The 10-year datasets from the trials of first-line anti-cytotoxic T lymphocyte-associated antigen-4 (CTLA4) therapy in terminal metastatic melanoma patients show an unprecedented long-term survival in 20% of patients [4].

The field was further ignited by the development of monoclonal antibodies targeting a second checkpoint the programmed death 1 (PD1)-programmed death-ligand 1 (PD-L1) axis [5]. A slew of antibodies have now entered clinical trials. PD-L1 expression may be useful as a biomarker, especially in the context of high PD-L1-expressing tumors, but presents several challenges. There are a variety of commercially available probes, all from the hybridomas of various animal species, and they each have different binding epitopes and differing performance on formalin-fixed, paraffin-embedded (FFPE) tissue, just to name a few technical challenges. Eventually, the authors of this editorial hope to see the use of PD-L1 as a biomarker streamlined internationally. Additionally, a clear understanding of individual disease biology and the differing mechanisms of action of immune checkpoints across various diseases will drive our knowledge further. There has been a large push to explore these drugs not only alone but also in combination with other tools available in the medical oncology clinic across many cancers. There is a clear need to treat all these studies as opportunities to understand in greater depth the interaction between the immune system and cancer. We have seen historically unprecedented sample sizes in early-phase clinical trials. As prospective trials continue to be formulated and run, we need to look retrospectively and study differences between responders and non-responders to understand how and why these checkpoints work [6].

Some early investigators have already done extensive research to understand immune checkpoint pathways within specific diseases. Margaret Shipp’s group has been studying 9p24 chromosomal aberrations in Hodgkin’s lymphoma for years, and through scrutiny identified PD-L1 up-regulation as an important factor in this disease [7]. Unsurprisingly, the application of anti-PD1 in Hodgkin’s disease has led to dramatic responses, the highest across any cancer type; even in the setting of post-transplant patients, it is effective for the patients who have a new donor immune system [8].

The development of chimeric antigen receptor (CAR) T cells is another truly exciting story in the annals of cancer treatment, and it is important to understand that the field has developed rapidly in a single peculiar context, B cell lymphomas and acute leukemias. There are very few known proteins that are cancer-specific rather than ubiquitous. CD19 is a key target of an intermediate form of B cells and humans can survive without this subset of CD19-positive B cells. Hence CD19 represents a useful cancer target. The most promising CAR T cells today target CD19 with dramatic responses seen in patients already refractory to multiple prior treatments [9, 10]. In July 2017, the United States Food and Drug Administration (FDA) voted unanimously 10 to 0 to approve the Novartis CAR-T cell CTL019. Hence, today, the two most mature, clinically advanced and possibly impactful cancer immunotherapies, monoclonal antibodies (mAb) targeting the immune checkpoint axes CTLA-4 and PD1-PD-L1 and adoptive transfer of genetically modified T cells expressing the CAR, have opened up a new paradigm and era in cancer treatment.

## Targeting the “Soil” to kill the “Seed”

Since 2014, the United States Food and Drug Administration (FDA) and some other drug regulatory agencies have approved PD1 signal inhibitors for advanced malignant melanoma, non-small cell lung cancer (NSCLC), Hodgkin’s lymphoma (the most stunning single agent responses of any cancer to date), renal cell cancer, bladder cancer, Merkel cell cancer, and squamous cell head and neck cancer following landmark clinical trials [5]. While PD1 signal inhibitors are generally safe and tolerable, their mechanistic action of “breaking” immune tolerance in the tumor microenvironment to unleash an endogenous anti-cancer adaptive immune response can uncommonly result in autoimmune complications, which include interstitial pneumonitis, colitis, type 1 diabetes mellitus, skin reactions, immune thrombocytopenia, neutropenia, encephalopathy, Guillain–Barre Syndrome, myasthenia gravis, myelitis, myocarditis, adrenalitis, thyroiditis, and nephritis [5]. Most of such rare immune toxicities occur within the first 4 months of starting treatment. Adverse effects caused by anti-PD1 antibodies are significantly less frequent and severe than adverse effects caused by anti-CTLA4 monoclonal antibody that had been approved earlier in 2011 for the treatment of advanced malignant melanoma [5]. Combining anti-CTLA4 antibody (ipilimumab) with anti-PD1 antibody (nivolumab) in advanced melanoma demonstrated more rapid, durable responses and improved overall survival compared with ipilimumab or nivolumab alone, which resulted in FDA accelerated approval of this combination in 2015 [11]. However, the immune-oncology therapy combination added grade III and IV toxicities. Pseudoprogression, where the tumor size grows larger before shrinking following treatment with immune checkpoint inhibitor, is rarely seen, except in melanoma. A more recent clinical puzzle is hyperprogression, where PD1 inhibitors may speed up tumor growth, reported in 9% of cancer patients treated with PD1 inhibitors in one series [12]. These patients tend to be older, possessing extra copies of the rare cancer-driving genes mouse double minute 2 homolog (*MDM2*) and (*MDM4*) [12]. Never seen before with other cancer treatment modalities, immune-oncology therapy can give a new lease of life to select incurable patients, called “supersurvivors,” who achieve durable responses and are alive even 10 years following immune-oncology treatment [4]. Since the first FDA approval of PD1 inhibitor in 2014, there are now over 800 clinical trials with PD1 signal inhibitors worldwide in progress [5].

Combining therapies rationally is the obvious logical strategy to maximize benefit, including combining PD1 inhibitors with radiation or chemotherapy. An emerging targeted therapy that enhances PD1 inhibitor potency is the treatment with mitogen-activated protein kinase (MEK) inhibitors, being able to convert a less immunogenic “cold” cancer into a more immunogenic “hot” cancer [13]. However, there should be caution in choosing combinations. For example, combining PD-L1 mAb durvalumab with epidermal growth factor receptor (EGFR) inhibitor osimertinib results in more interstitial pneumonitis [14].

Can predictive biomarkers identify patients for better PD1 inhibitor treatment? The FDA has approved PD-L1 expression by immunohistochemistry (IHC) as a diagnostic companion for PD1 inhibitor therapy. There has been concern about the variability in PD-1 expression as a result of varying performance of various IHC PD-L1 antibody. So far the value of measuring PD-L1 expression is still controversial, though it appears that the amount of CD8 T cells around the tumor, the closer to the tumor the better, can augment the predictive value of PD-L1 expression as an efficacy biomarker [6]. Mutational burden of a cancer is an important biomarker for PD1 inhibitor success [15]. In DNA mismatch repair (MMR)-deficient colorectal cancer, the response to PD1 inhibition is far superior versus MMR-proficient cancer, confirming the hypermutated immunogenic status of these MMR-deficient cancers [16]. This has led to an FDA approval of a test for microsatellite instability representing the tumor’s mutational burden to more precisely guide PD1 inhibitor success across all cancer types [17]. Beyond MMR-deficient status representing microsatellite instable tumors (MSI), a recent study suggests that a composite score summarizing density of memory and cytotoxic T cells could better predict patient survival as compared to the microsatellite instability alone [18].

## T cells or not T cells

The world of cancer immunotherapy today, given the space in which advances have been seen, is rather T cell-centric; not just CAR T cells, but specific antigen-targeting T-cell receptor transduced T cells and tumor-infiltrating lymphocytes expanded to express specific mutated neoantigen targeting the oncogenic signalling molecules such as KRAS in colorectal cancer [19]. We now have emerging studies exploring the role of immune checkpoints in other cell types too, such as CD47 in macrophages, and CD137 in natural killer cells [20, 21]. Ultimately, it is most likely that a concerted effort to systematically drive advances forward will be required across all cancer subtypes. In our search for biomarkers of single drug therapy, and subsequently multiple drug combinations, there is a clear need to address some fundamental questions. First, do patients intrinsically have immune cells that recognize cancer? Second, do these immune cells exert tolerogenic effects or are they able to elicit meaningful anti-cancer responses? What are the barriers to any anti-cancer immune response with regards to the tumor microenvironment, and are factors such as pH, or hypoxia, or glucose transport programs already in place to abrogate any possible immune response? Is there a kinetic failure with regards to the immune system? Is it possible to confer increased immunogenicity to the tumor microenvironment, ranging from radiation, thermal, photodynamic, and conventional systemic therapy agents that now include chemotherapy, small molecule inhibitors, and antibodies? Critically, is the cancer feasibly targeted using a single protein-directed therapy or are there obstructing challenges with tumor heterogeneity that prevent this from being a durable strategy? Finally, what are escape pathways that are likely for resistance to cancer immunotherapy? It may be that a confluence of factors may have led to malignant melanoma being the premise for the initial success of immune checkpoint inhibitors, as this was a disease recognized to have high mutational burden, with a higher chance of immunogenic neoantigens being presented. In any case, this disease has a high percentage of tumor-infiltrating lymphocytes.

While the explosion of cancer immunotherapy studies has been thoroughly explored in western populations, there is a paucity of data emerging from Asian populations. Compared with western/Caucasian populations, Asians have distinct human leukocyte antigen (HLA) types, genotype, phenotype and microbiome. Asians also have distinct cancers rarely seen in the West. Some examples include Epstein-Barr virus (EBV)-related cancer, rarely seen in western populations, and acral lentiginous melanomas that is very distinct from melanoma that is attributed to sun exposure.

## Back to the future

The remarkable expansion of preclinical and clinical studies on cancer immunotherapy in only the last few years has been stunning. On the other hand, barriers to further development and broad application are already apparent. Response rates with PD1 inhibitors in many solid tumors generally appear to plateau at approximately 20% with modest overall survival benefits while CAR T-cell therapy appears to have hit a brick wall in terms of success in solid tumors. Spiralling costs of such therapies are prohibitive and thus unsustainable, and this issue is further augmented by the prospect of a future of expensive drug combinations. Is this the beginning of the end for cancer immunotherapy? The “*Weizexi”* incident in China has prompted the China FDA to review cell therapy regulations more closely and the Chinese Society for Clinical Oncology to establish a cancer immunotherapy committee specifically to advance the field systematically—a good thing from which will emerge stronger scientific and clinical studies. The recent deaths in the United States of cerebral edema and cytokine release storm using CAR T-cell therapy following preconditioning chemotherapy has also evoked serious scrutiny and caution from regulators, academic medical centers, and the industry alike. There are currently over 160 clinical trials of T-cell therapy worldwide, with 102 CAR T-cell trials alone. Surely, technological advances will shorten cell production time and improve anti-cancer efficacy. For example, a Chinese group has used the clustered regularly interspaced short palindromic repeat (*CRISPR*)-*Cas9* gene editing system to engineer CAR T cells to disable its PD1 so as to enhance the adaptive T-cell immune attack in an ongoing safety-focused pilot clinical trial (ClinicalTrials.gov NCT02793856). Cellectis has employed another gene editing technique called transcription activator-like effector nucleases (*TALEN*) to produce off-the-shelf allogenic CAR T cells that is “invisible” to the host immunity to maximize its anti-cancer effect [22]. The global markets remain optimistic, forecasting a market size of USD $6 billion for T-cell therapy by 2025, and USD $34 billion for immune-oncology therapy by 2024 [23, 24]. Moving forward, precision cancer immunotherapy will allow for higher success for individualized treatment via predictive cancer-immune biomarker systems such as the *cancer immunogram* [25]. This can also further improve design of rational combination therapies and optimize benefit.

Today, Asia is home to over 4 billion people (60% of the world’s population) in almost 50 countries of diverse genetic and phenotypic heterogeneity, and the most rapidly ageing in the world [26]. A quarter of all cancer deaths in the world are in China, as described by Professor Paul Goss of Harvard Medical School [26]. The cancer incidence in China is 264.85/100,000; that is, China has nearly 3 million new cancer cases per year [27]. Therefore, more cancer immunotherapy trials should be conducted in Asia, as its cancer burden increases to a staggering 163 per 100,000 people by 2030 [26]. In the new vista of cancer immunotherapy and its global impact on the lives of patients, this is not the beginning of the end, but only the end of the beginning.

## Abbreviations

CAR: chimeric antigen receptor
mAb: monoclonal antibodies
FDA: Food and Drug Administration
NSCLC: non-small cell lung cancer
EGFR: epidermal growth factor receptor
IHC: immunohistochemistry
MMR: mismatch repair
MSI: microsatellite instable tumors
